# Independent and combined impact of hypoxia and acute inorganic nitrate ingestion on thermoregulatory responses to the cold

**DOI:** 10.1007/s00421-021-04602-x

**Published:** 2021-02-09

**Authors:** Josh T. Arnold, Stephen J. Bailey, Simon G. Hodder, Naoto Fujii, Alex B. Lloyd

**Affiliations:** 1 Environmental Ergonomics Research Centre, James France Bldg, Design School, Loughborough University, Loughborough, LE11 3TU UK; 2 School of Sport, Exercise and Health Sciences, Loughborough University, Loughborough, UK; 3 Faculty of Health and Sport Sciences, University of Tsukuba, Tsukuba, Japan

**Keywords:** Cold, Hypoxia, Nitric oxide, Shivering, Vasoconstriction, Anapyrexia

## Abstract

**Purpose:**

This study assessed the impact of normobaric hypoxia and acute nitrate ingestion on shivering thermogenesis, cutaneous vascular control, and thermometrics in response to cold stress.

**Method:**

Eleven male volunteers underwent passive cooling at 10 °C air temperature across four conditions: (1) normoxia with placebo ingestion, (2) hypoxia (0.130 *F*_*i*_O_2_) with placebo ingestion, (3) normoxia with 13 mmol nitrate ingestion, and (4) hypoxia with nitrate ingestion. Physiological metrics were assessed as a rate of change over 45 min to determine heat loss, and at the point of shivering onset to determine the thermogenic thermoeffector threshold.

**Result:**

Independently, hypoxia expedited shivering onset time (*p* = 0.05) due to a faster cooling rate as opposed to a change in central thermoeffector thresholds. Specifically, compared to normoxia, hypoxia increased skin blood flow (*p* = 0.02), leading to an increased core-cooling rate (*p* = 0.04) and delta change in rectal temperature (*p* = 0.03) over 45 min, yet the same rectal temperature at shivering onset (*p* = 0.9). Independently, nitrate ingestion delayed shivering onset time (*p* = 0.01), mediated by a change in central thermoeffector thresholds, independent of changes in peripheral heat exchange. Specifically, compared to placebo ingestion, no difference was observed in skin blood flow (*p* = 0.5), core-cooling rate (*p* = 0.5), or delta change in rectal temperature (*p* = 0.7) over 45 min, while nitrate reduced rectal temperature at shivering onset (*p* = 0.04). No interaction was observed between hypoxia and nitrate ingestion.

**Conclusion:**

These data improve our understanding of how hypoxia and nitric oxide modulate cold thermoregulation.

## Introduction

Across taxa, it is well documented that internal body temperature is positively associated with oxygen uptake (V̇O_2_) (Krogh 1914; Wood 1991). In situations of systemic hypoxia, the thermoregulatory tendency is that of a reduction in body temperature (anapyrexia), with an accompanying reduction in V̇O_2_, improving metabolic efficiency (Steiner and Branco 2002). However, a decline in skin and deep body temperature elicit robust thermogenic responses, the most effective of which is shivering (Stocks et al. 2004). Since maximal shivering can reach up to 40% V̇O_2max_ (Eyolfson et al. 2001), this response might be considered metabolically costly under limited oxygen availability. Thus, it is conceivable that the competing demands of cold and hypoxia compromise the ability to maintain both thermal balance and adequate oxygenation when both stimuli are simultaneously encountered (Wood 1991).

Considering peripheral mechanisms through which cold and hypoxia interact, nitric oxide (NO) production appears to mediate hypoxia-induced vasodilation (Steiner and Branco 2002; Umbrello et al. 2013), specifically in the cutaneous microvasculature (Arnold et al. 2020b), while NO suppression is fundamental to cold-induced local cutaneous vasoconstriction (Johnson et al. 2014). The extent to which NO attenuates noradrenaline induced cutaneous vasoconstriction, if at all, remains unclear (Shibasaki et al. 2008; Arnold et al. 2020b). In humans, hypoxic peripheral vasodilation translates to increased skin temperatures (Blatteis and Lutherer 1976) and lower core temperatures (Cipriano and Goldman 1975) when cold exposure is combined with hypoxia compared to normoxia. Subsequently, greater heat loss with combined cold and hypoxia leads to earlier shivering onset (Johnston et al. 1996) and a faster progression from intermittent to sustained shivering (Blatteis and Lutherer 1976).

Considering central mechanisms, while cold thermoeffectors remain operational in low oxygen conditions, central nervous system (CNS) hypoxia appears to reduce body temperature via altered thermosensitivity (afferent processing), neural activation (efferent drive), and ultimately the thermoeffector threshold—defined in the present study as the core temperature at which shivering occurs (Tattersall and Milsom 2009). Hypoxia-driven NO synthesis appears fundamental to elicit central effects (Branco et al. 1997; Steiner and Branco 2002), with two mechanisms proposed: (1) NO increases the activity and thermosensitivity of warm-sensitive neurons in the preoptic anterior hypothalamus, leading to an ‘overestimation’ of the integrated thermal signal, and inhibition of cold-sensitive neurons (Boulant 2000), thus a reduction in cold thermoeffector thresholds; and (2) NO reduces sympathetic tonus across brain regions, lowering the firing rate of sympathetic fibres, in turn reducing thermogenic neural drive and vasoconstriction across vascular beds (Steiner and Branco 2002). Yet, given that most hypoxic-anapyrexia investigations have been carried out in small mammals, the translation of central mechanisms to humans should be interpreted with caution. Indeed, humans directly regulate against hypoxia—‘oxyregulators’, possibly alleviating the need for a reduction in body temperature, whereas small mammals generally respond to hypoxia via secondary means—‘oxyconformers’, for example altering body temperature (Gu and Jun 2018). Furthermore, hypothalamic thermosensitivity scales negatively with body mass, while thermosensitivity in large mammals is mediated via comparable contributions of central and peripheral inputs; in small mammals, it is likely mediated solely by central inputs (Tattersall and Milsom 2009). Hypoxic modulation of central thermoregulatory mechanisms requires elucidation in human subjects.

Summarising human investigations to date, it remains unclear if and how hypoxia definitively alters cold thermoregulation in humans (Gu and Jun 2018). A number of studies report compromised thermal responses to the cold with hypoxia (Blatteis and Lutherer 1976; Robinson and Haymes 1990; Johnston et al. 1996; DiPasquale et al. 2015), while others show little/no effect (Simmons et al. 2011; Keramidas et al. 2014; O’Brien et al. 2015; Seo et al. 2017). No study has explored the interaction of hypoxic and cold stress and its modulation by altered NO availability in humans, whether through peripherally or centrally mediated aforementioned mechanisms. While investigation of NO activity via systemic NO synthase (NOS) inhibition is methodologically complex, a convenient, safe, and non-invasive approach to increase NO bioavailability in humans is dietary supplementation with inorganic nitrate (NO_3_^−^) (Lundberg and Weitzberg 2010). Noteworthy, the reduction of NO_3_^−^ through to NO is potentiated in hypoxia (Modin et al. 2001; Castello et al. 2006); however, the direct effect of dietary NO_3_^−^ supplementation on thermoregulatory responses to the cold, in addition to its modulation of the thermoregulatory response to combined cold and hypoxia, has yet to be determined.

This study assessed the impact of independent and combined normobaric hypoxia and acute dietary nitrate ingestion on shivering thermogenesis and vascular control in response to whole-body cooling. Two hypotheses were examined, as a rate of change over 45 min, serving as a proxy for peripheral heat exchange, and at the point of shivering onset, serving as a proxy for thermogenic thermoeffector thresholds: (1) over 45 min, hypoxia would blunt peripheral vasoconstriction, leading to an increase in skin blood flow and rate of heat loss compared to normoxia, with this response synergised following concomitant nitrate ingestion; (2) at shivering onset, no difference in skin or core temperature would be observed between hypoxia and normoxia, yet faster heat loss with hypoxia would result in a temporally earlier shivering onset, with this response further synergistically accelerated following concomitant nitrate ingestion. A better understanding of how hypoxia modulates thermoregulation, and the underpinning role of NO, could aid in the development of interventions and countermeasures for use in clinical settings; for example, improving the use and understanding of cold therapy to offset the effects of hypoxia, possibly with increased efficacy via dietary nitrate ingestion.

## Methods

### Participants

Eleven healthy male volunteers (age, 23 ± 2 years; stature, 1.80 ± 0.06 m; body mass, 74.9 ± 9.4 kg; BMI, 23 ± 2 kg·m^2^; body fat, 14 ± 3%) were recruited from the Loughborough area, UK between January and November 2019. All participants were physically active and over 18 years of age. Exclusion criteria included smokers and any individuals with a history of muscular, neurological, or cardiovascular debilities. Females were excluded from the study due to the difficulty in controlling for menstrual phase across four experimental visits, separated by wash-out days, since female hormones alter reflex cutaneous vasoconstriction during cold exposure (Stephens et al. 2002). Participants provided written informed consent before participating. Ethical approval was granted by the Ethics Committee at Loughborough University (approval number, R18-P189). Research was conducted in accordance with the Declaration of Helsinki, 2013, except for registration in a database.

### Study design

The study utilised a single-blind repeated-measures randomised design. Participants were exposed to a standardised whole-body cooling stimulus, across five sessions; a familiarisation session, followed by four experimental trials involving independent and combined normobaric hypoxic stress and acute oral nitrate administration: **NM_PLC**, normoxia with placebo ingestion; **HYP_PLC**, hypoxia with placebo ingestion; **NM_NTR**, normoxia with NO_3_^−^ ingestion; **HYP_NTR**, hypoxia with NO_3_^−^ ingestion. A minimum 24 h wash-out period was ensured between sessions to allow circulating nitrite [NO_2_^−^] to return to basal levels (Wylie et al. 2013), and eliminating any order effect or adaptation in response to the cold (Arnold et al. 2020a). Sessions commenced at the same time each day to exclude the extraneous impact of circadian rhythms upon thermoregulation, as previously observed (Kondo et al. 2009).

To elicit uncompensable whole-body cooling, participants rested supine in an air-conditioned room with cold air circulation, wearing shorts and socks (air temperature, 9.6 ± 0.6 °C; relative humidity, 57 ± 4%; air velocity, 0.42 ± 0.17 m·s^−1^). To minimise thermal insulation of the underside of the body by the bed, participants laid on a water-perfused mattress circulating water at 25 °C (PlastiPad®, Gentherm, OH, USA). The temperature of the mattress was selected to provide a comparable cooling rate as the ambient air, confirmed during pilot work using a thermal imaging camera. Normobaric hypoxic inspired air (F_i_O_2,_ 0.130), equivalent to ~ 3750 m above sea level as used previously (Robinson and Haymes 1990; Keramidas et al. 2014; DiPasquale et al. 2015; Seo et al. 2017), was administered via a face mask and Hans Rudolf three-way valve, connected to a series of pre-filled Douglas bags via low-resistance silicon pipe. During normobaric normoxic trials (F_i_O_2,_ 0.209), Douglas bags were filled with sea-level air for placebo purposes.

Oral NO_3_^−^ was administered as two 70 mL concentrated beetroot juice shots which were either rich (~ 13 mmol NO_3_^−^, Beet It Sport™, James White Drinks Ltd, UK) or low (~ 0.003 mmol NO_3_^−^, James White Drinks Ltd, UK) in NO_3_^−^. Previous research shows physiological efficacy of NO_3_^−^ doses in the range of 8.4–16.8 mmol (Wylie et al. 2013; James et al. 2015). Placebo shots were identical in appearance, taste, and texture, created by passing the NO_3_^−^ active beetroot juice through a Purolite A520E NO_3_^−^ selective ion-exchange resin prior to pasteurisation (Lansley et al. 2011). Checking that the respective placebo conditions had been theoretically effective, a manipulation check was conducted after each visit, asking participants to guess what experimental conditions they had received.

### Procedure

**Fig. 1:**
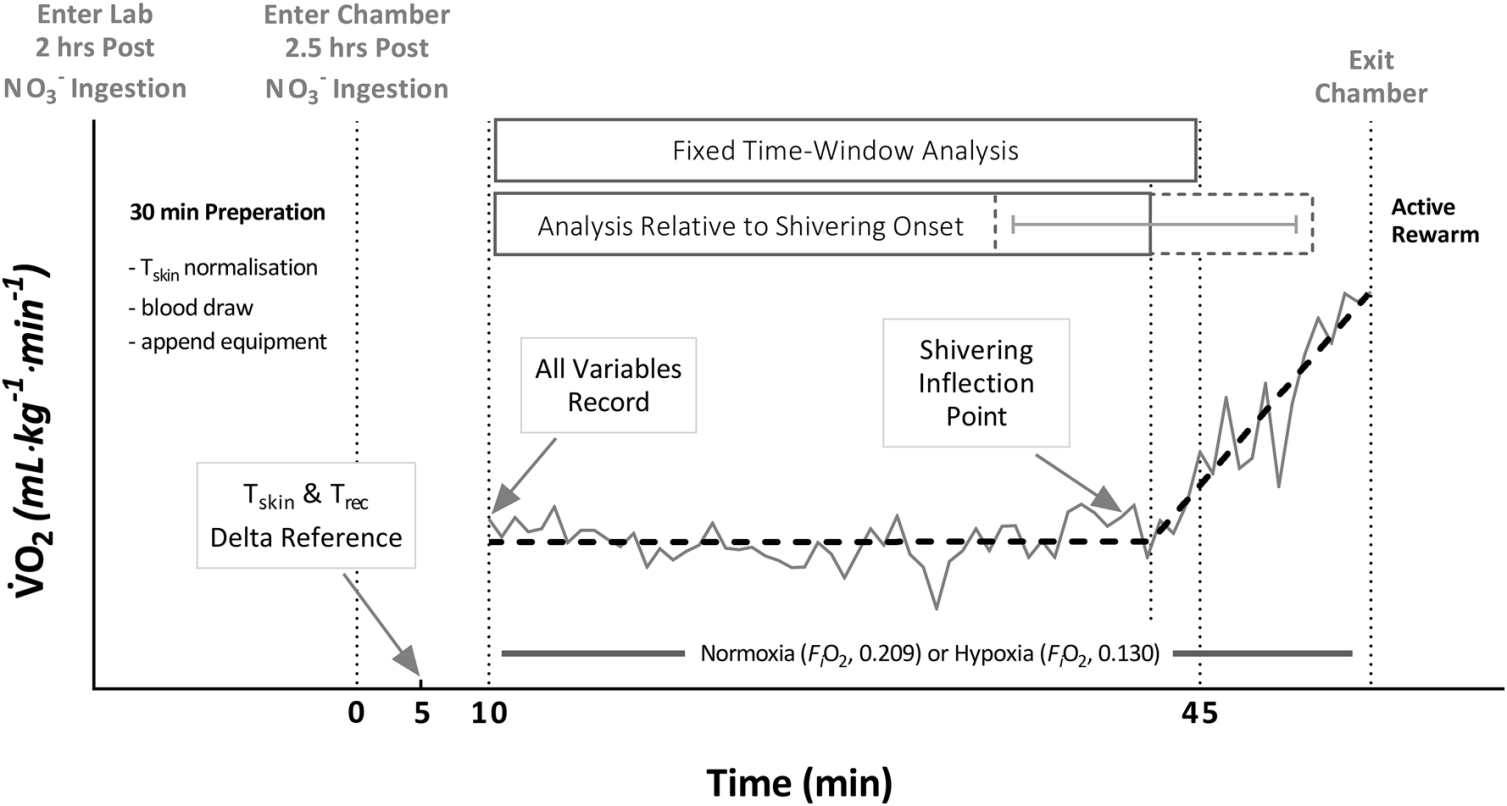
Schematic representation of the experimental protocol with sample whole-body oxygen consumption output from a representative individual. A single-blind repeated-measures design in which 11 participants visited the laboratory on four occasions, 2 h post-ingestion of either nitrate (NO_3_^−^) rich beetroot juice or a nitrate depleted beetroot juice placebo. Participants underwent whole-body cooling in an air-conditioned room, resting supine on a water-perfused mattress (temperature: air, 9.6 °C; bed, 25.0 °C). During cooling, participants inspired either normoxic or hypoxic air via face mask. * T*_skin_, skin temperature, * T*_rec_, rectal temperature, *F*_*i*_O_2_, faction of inspired oxygen

An overview of the procedure is presented in Fig. 1. Following an initial study briefing, participants attended a familiarisation session involving the same cooling protocol as experimental trials (detailed below). At the end of the session, participants were reminded to refrain from alcohol and any non-routine vigorous activity 24 h preceding each subsequent trial, hydrate ad-libitum, and avoid caffeine 6 h prior to each trial. Importantly, participants were also asked to abstain from the use of antibacterial mouthwash as this has previously shown to thwart the reduction of NO_3_^−^ to NO_2_^−^ by commensal bacteria in the oral cavity (Govoni et al. 2008). The relevant shots were allocated, along with a sachet of porridge, ready for ingestion prior to the next session.

On the experimental trial day, shots and porridge were ingested at home 2 h prior to arrival at the laboratory. A rectal thermistor (400-AC Temperature Probe, Viamed Ltd., UK) was first provided upon arrival, self-administered 100 mm beyond the anal sphincter (European Committee for Standardization 2004). A urine sample was also taken at this point to ensure adequate hydration; urine-specific gravity (USG) less than 1.020 (Oppliger et al. 2005). Body mass and stature were then assessed, after which the participant rested seated in a thermoneutral prep-room, allowing skin temperature to normalise and measurement probes to be appended. At 2 h 15 min post-ingestion, a single 6 mL venous blood sample was drawn from the antecubital fossa into a lithium heparin tube (Vatcutainer™, BD, USA). Blood samples were immediately centrifuged at 4000 rpm and 4 °C for 10 min. Plasma was aliquoted into 1.5 mL microtubes and frozen at − 80 °C for subsequent analysis of [NO_2_^−^] using ozone-based chemiluminescence as per Wylie et al. (2013).

At 2 h 30 min post-NO_3_^−^ ingestion, the participant was transported through to the temperature controlled experimental room where whole-body cooling commenced. Instruction was given to adopt a supine position on the water-perfused mattress, at which point the trial began. Participants were encouraged to relax, remain still, while avoiding any behavioural thermoregulation. Inspired air was administered from Douglas bags at 5 min, which contained either hypoxic or normoxic air. Allowing time to connect the remaining apparatus, all physiological variables were set to record at 10 min. Participants were progressively cooled for minimum period of 45 min, beyond which the trial continued until 10 min after the participant was deemed to be exhibiting continuous whole-body shivering via visual assessment. Upon completion of the cold exposure, participants were removed from the cold and actively re-warmed on a cycling ergometer. A hot shower was offered to restore all remaining thermal comfort, before participants were allocated their next beetroot juice shots and porridge ready for consumption prior to the next trial.

### Measurements

Physiological metrics were continually assessed during whole-body cooling through until the point of trial termination. Mean skin temperature was assessed via surface thermistors (Grant Instruments, UK), fixed in place with Hypafix® tape (BSN medical, UK), with an equal weighting taken from 14 sites as per ISO 9886 (European Committee for Standardization 2004). Skin and rectal thermistors fed into a data logger (SQ2020, Grant, UK), sampling every 1 s. Skin blood flow was assessed via multi-fibre array laser Doppler probe (VP1T/7, Moor Instruments, UK) sampling every 1 s at the inner right forearm. The laser Doppler probe was carefully positioned ensuring repeatability across trials. Cutaneous vascular conductance (CVC) was also assessed by dividing laser Doppler flux by the closest temporal measurement of mean arterial pressure ([1/3 systolic blood pressure] + [2/3 diastolic blood pressure]) via automated sphygmomanometer (Tango M2, SunTech Medical, USA), sampling every 5 min from the contralateral arm. Heart rate via three-lead electrocardiogram and peripheral oxygen saturation (S_p_O_2_) via pulse oximeter were assessed every 5 min (Tango M2). Expired respiratory variables were assessed via metabolic cart (Quark CPET, Cosmed, ITL) with a two-point calibration prior to each trial and connected via mixing chamber to the expiratory side of a Hans Rudolf valve attached to a face mask. Finally, shivering thermogenesis was continually assessed as per Arnold et al. (2020a) using pulmonary oxygen uptake (V̇O_2_), electromyography (EMG), via wireless surface electrode (DataLITE Wireless, Biometrics Ltd, UK) and mechanomyography (MMG), via tri-axial accelerometer (NXP Semiconductors, NL). Both EMG and MMG sensors were placed on the right-hand side of the body, over the centre of the muscle belly, with an MMG sensor positioned at the right pectoralis major, and EMG sensors positioned at the pectoralis major, deltoid and trapezius. Mean EMG was established, taking an equal weighting from all sites. Diligence was paid in relation to the reproducibility of sensor placement and the preparation of the skin in accordance with SENIAM recommendations (Hermens et al. 1999).

### Data analysis

All variables were collated into 1 min time block averages for comparison across conditions, while raw V̇O_2_, EMG and MMG data were also processed as per Arnold et al. (2020a) for the quantification of shivering onset via inflections in data over time. To increase the precision in the estimate, shivering onset for any given trial was categorised as the mean of six observations; the time elapsed at the inflection point in V̇O_2_, EMG, and MMG data, each quantified by both segmental linear regression (free from observer bias) and visual inspection of graphs. The coefficient of variation between these six observations was 8%, and thus, using the mean of all six inflection point observations was deemed necessary compared to any one specific observation.

Further comparisons across experimental conditions were made in two ways (Fig. 1): (1) first, across a discrete 10 min–45 min fixed time period, shared by all participants and trials. Variables were considered as a mean value across this period, with exception of skin and rectal temperatures which were considered as delta (Δ) changes, to minimise the influence of any day-to-day variation. A core-cooling rate was also established, defined as the slope of the relationship between rectal temperature and time. (2) Second, across a variable 10 min to shivering onset time period, individualised to each participant in any one specific trial. Again, variables were considered as a mean across this period, while skin and rectal temperatures were considered as delta (Δ) changes.

### Statistical analysis

Inferential statistical analysis was conducted using the software package IBM SPSS Statistics for Windows (vs. 25, IBM Corp., USA). Main effects for hypoxia and nitrate ingestion, and their associated interaction were assessed via two-way repeated-measures ANOVA (2 × 2). Where no main effect was observed for a factor, interactions were not explored further. Correlations of primary variables (shivering onset, CVC, skin temperature, and rectal temperature) with mean S_p_O_2_ or plasma [NO_2_^−^] were investigated via Pearson’s correlation test. Alpha was set a priori at 0.05. Interactions are defined as per Lloyd and Havenith (2016). Data are presented as mean ± SD; unless otherwise stated, main effects are presented as collapsed estimated marginal means (EMM) with Bonferroni adjusted confidence intervals [95% CI’s].

## Results

No differences were observed in hydration status across trials (USG, *p* = 0.2). Furthermore, no differences were observed in either skin temperature (*p* = 0.7), rectal temperature (*p* = 0.3), mean arterial pressure (*p* = 0.3), or heart rate (*p* = 0.06) at the initiation of whole-body cooling across trials. Experimental stressors were effective in their intended application; hypoxia elicited a significant reduction in peripheral oxygen saturation compared to normoxia [EMM; normoxia, 99 (98–99) % vs. hypoxia, 88 (85–90) %, *p* < 0.001], while nitrate ingestion elicited a significant increase in plasma [NO_2_^−^] compared to the placebo [EMM; placebo, 117 (82–151) nmol·L^−1^ vs. nitrate, 575 (394–757) nmol·L^−1^, *p* < 0.001]. The manipulation check indicated that 2 of the 11 individuals correctly guessed their respective trial order, 4 individuals incorrectly guessed, and 5 individuals were unable to distinguish their trial order.

### Primary variables

**Fig. 2:**
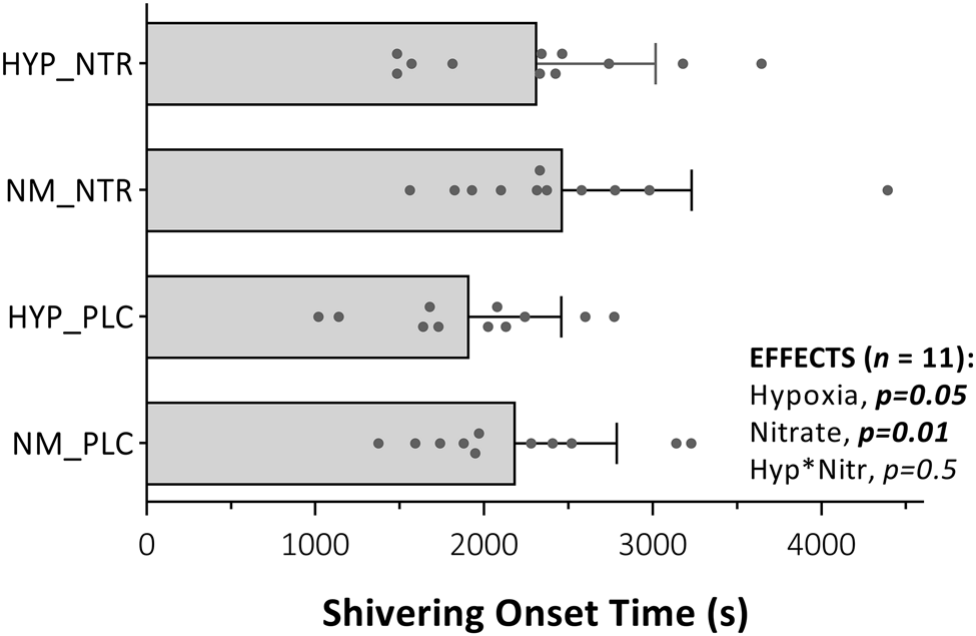
Onset of shivering thermogenesis in response to whole-body cooling with independent and combined hypoxia and acute inorganic nitrate ingestion. NOTE: ***NM_PLC***, normoxia (*Fi*O_2_, 0.209) with placebo ingestion (0.003 mmol NO_3_^−^); ***HYP_PLC***, hypoxia (*Fi*O_2_, 0.130) with placebo ingestion; ***NM_NTR***, normoxia with nitrate ingestion (13 mmol NO_3_^−^); ***HYP_NTR***, hypoxia with nitrate ingestion. Data are mean ± SD, with individual data points. Each data point was categorised as the mean of six observations; the time elapsed at the inflection point in whole-body oxygen uptake, electromyography, and mechanomyography data, each quantified by both segmental linear regression and visual inspection of graphs. Main effects (hypoxia and nitrate) and interaction (hypoxia × nitrate) assessed via two-way repeated-measures ANOVA (*α* = 0.05)

**Fig. 3:**
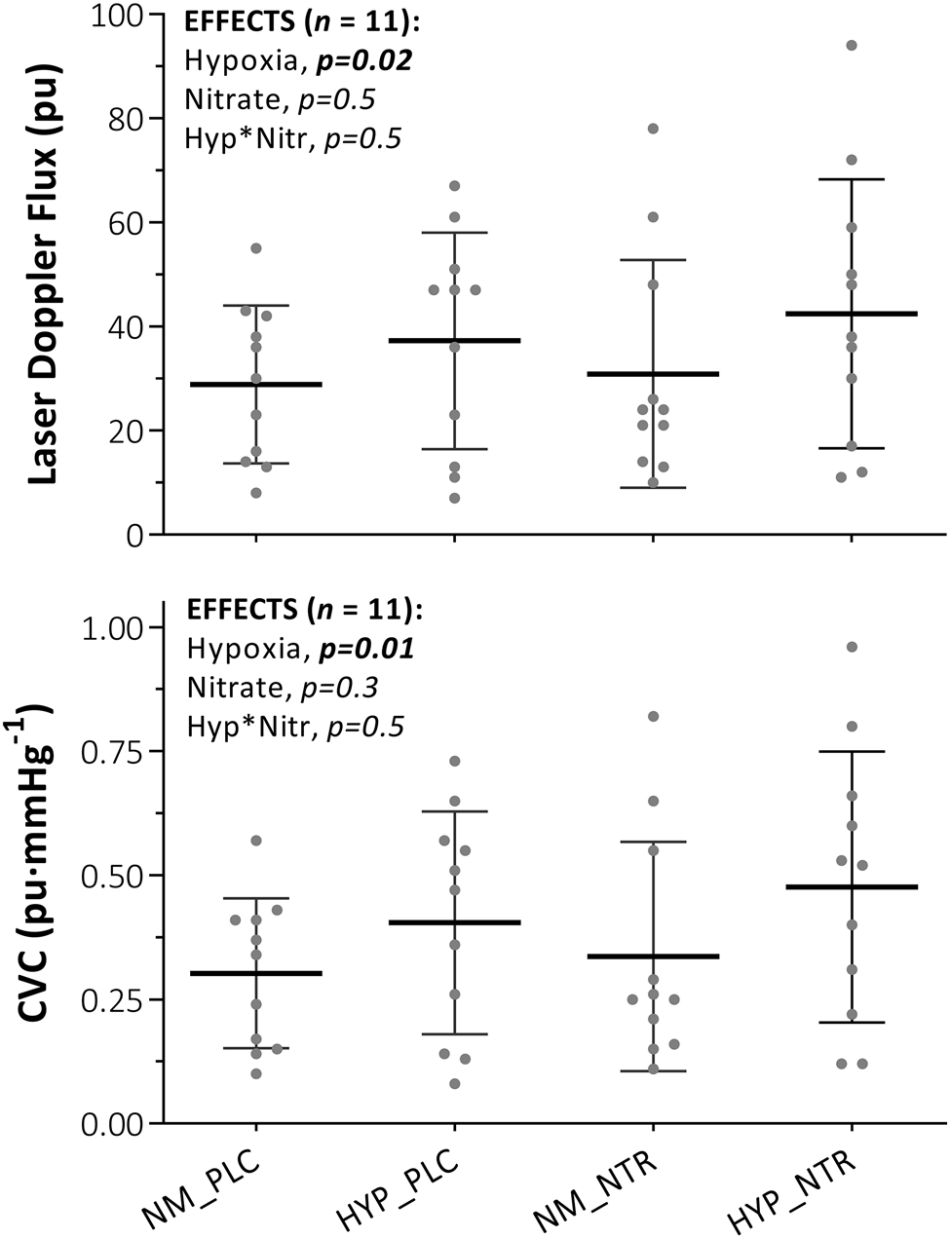
Skin blood flow and cutaneous vascular conductance in response to whole-body cooling with independent and combined hypoxia and acute inorganic nitrate ingestion. NOTE: ***NM_PLC***, normoxia (*Fi*O_2_, 0.209) with placebo ingestion **(**0.003 mmol NO_3_^−^**)**; ***HYP_PLC***, hypoxia (*Fi*O_2_, 0.130) with placebo ingestion; ***NM_NTR***, normoxia with nitrate ingestion **(**13 mmol NO_3_^−^); ***HYP_NTR***, hypoxia with nitrate ingestion. Variables were assessed across a fixed 10 min to 45 min time-window shared by all participants and trials. Data are mean ± SD, with individual data points. Main effects (hypoxia and nitrate) and interactions (hypoxia × nitrate) assessed via two-way repeated-measures ANOVA (*α* = 0.05)

Significant main effects were observed for both hypoxia and nitrate ingestion on shivering thermogenesis onset (Fig. 2); hypoxia decreased onset time [EMM; normoxia, 2329 (1907–2750) s vs. hypoxia, 2115 (1713–2517) s, *p* = 0.05], while nitrate ingestion increased onset time [EMM; placebo, 2052 (1690–2413) s vs. nitrate, 2392 (1925–2860) s, *p* = 0.01]. Hypoxia increased skin blood flow [EMM; normoxia, 30 (19–41) pu vs. hypoxia, 40 (25–54) pu, *p* = 0.02] and CVC [EMM; normoxia, 0.32 (0.20–0.44) pu·mmHg^−1^ vs. hypoxia, 0.44 (0.29–0.60) pu·mmHg^−1^, *p* = 0.01], assessed as the mean of values between 10 and 45 min, while nitrate ingestion showed no effect on skin blood flow [EMM; placebo, 33 (22–44) pu vs. nitrate, 37 (21–52) pu, *p* = 0.5] or CVC (EMM; placebo, 0.35 (0.24–0.47) pu·mmHg^−1^ vs. nitrate, 0.41 (0.25–0.57) pu·mmHg^−1^, *p* = 0.3] (Fig. 3). No interaction was observed between hypoxia and nitrate ingestion on either shivering onset, skin blood flow, or CVC (*p* = 0.5), and no correlation was observed between these variables and either peripheral oxygen saturation or plasma [NO_2_^−^] across any given condition (*p* > 0.09).

**Fig. 4:**
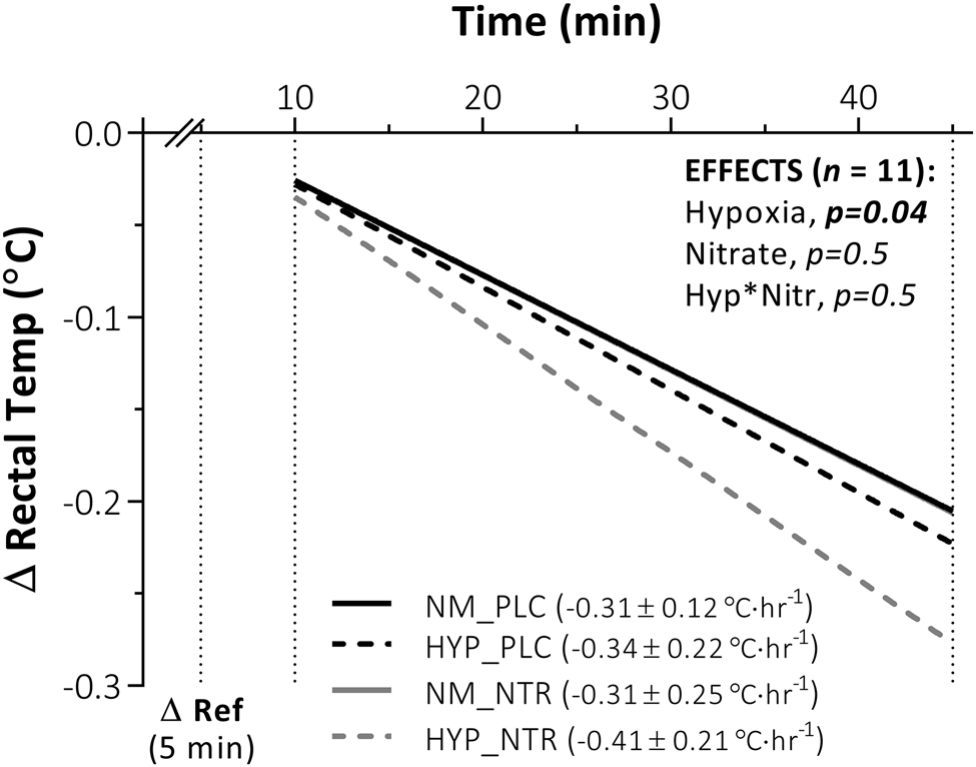
Core cooling rate in response to whole-body cooling with independent and combined hypoxia and acute inorganic nitrate ingestion. NOTE: ***NM_PLC***, normoxia (*Fi*O_2_, 0.209) with placebo ingestion **(**0.003 mmol NO_3_^−^**)**; ***HYP_PLC***, hypoxia (*Fi*O_2_, 0.130) with placebo ingestion; ***NM_NTR***, normoxia with nitrate ingestion **(**13 mmol NO_3_^−^); ***HYP_NTR***, hypoxia with nitrate ingestion. Rectal temperature considered as delta (Δ) changes from baseline. Main effects (hypoxia and nitrate) and interactions (hypoxia × nitrate) assessed via two-way repeated-measures ANOVA (*α* = 0.05). Noteworthy, NM_NTR is overlaid by NM_PLAC

**Fig. 5:**
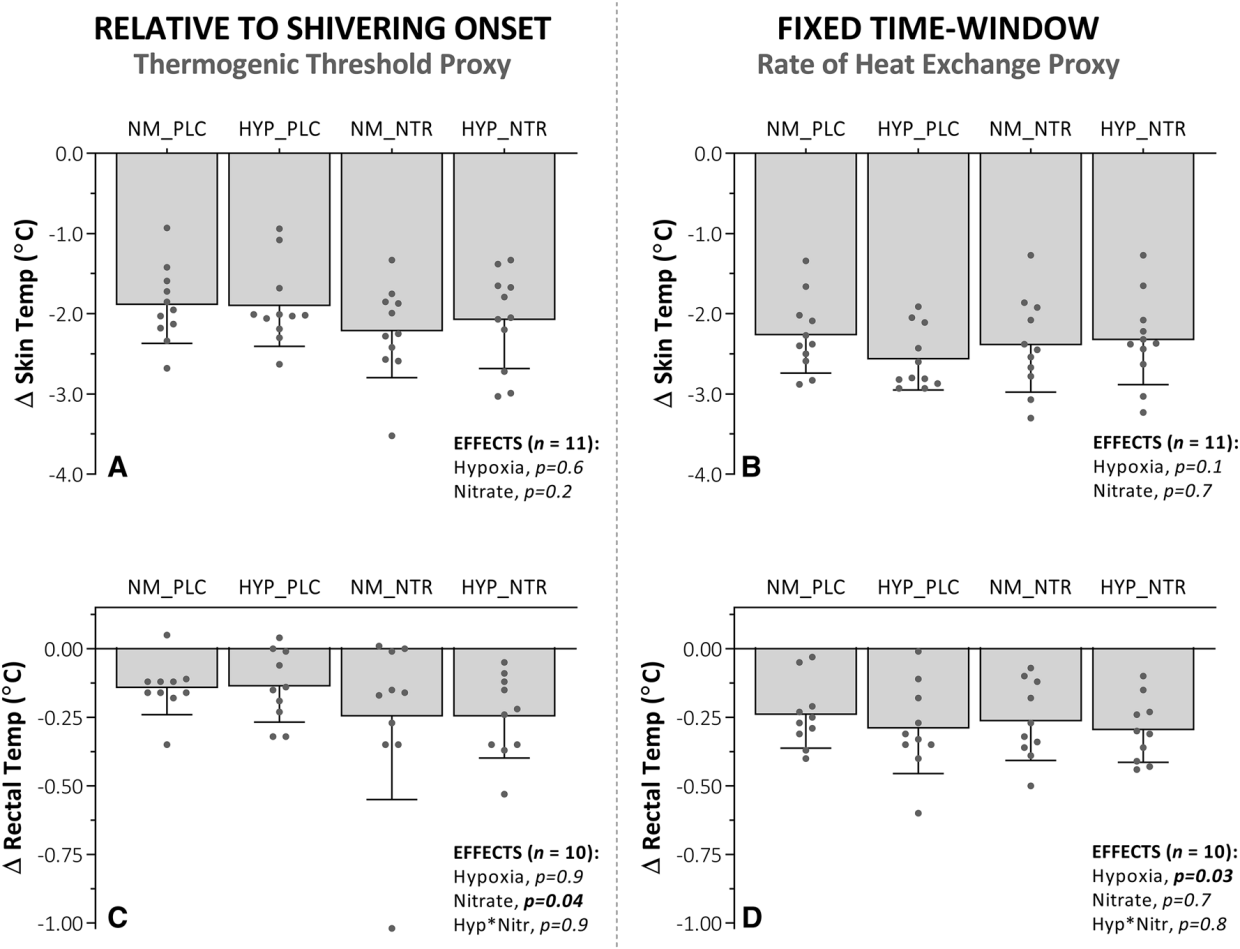
Thermometrics in response to whole-body cooling with independent and combined hypoxia and acute inorganic nitrate ingestion. NOTE: ***NM_PLC***, normoxia (*Fi*O_2_, 0.209) with placebo ingestion **(**0.003 mmol NO_3_^−^**)**; ***HYP_PLC***, hypoxia (*Fi*O_2_, 0.130) with placebo ingestion; ***NM_NTR***, normoxia with nitrate ingestion **(**13 mmol NO_3_^−^); ***HYP_NTR***, hypoxia with nitrate ingestion. Skin and rectal temperatures are considered as delta (Δ) changes from baseline, quantified at the end of a fixed 45 min time-window, shared by all participants and trials, serving as a proxy for the rate of heat exchange/loss (panels ***B*** and ***D***), and at shivering onset, serving as a proxy for the thermogenic thermoeffector threshold (panels ***A*** and ***C***). Data are mean ± SD, with individual data points. Main effects (hypoxia and nitrate) and interactions (hypoxia × nitrate) assessed via two-way repeated-measures ANOVA (*α* = 0.05)

Hypoxia increased core-cooling rate compared to normoxia [EMM; normoxia, − 0.31 (− 0.44 to − 0.18) °C·hr^−1^ vs. hypoxia, − 0.38 (− 0.51 to − 0.25) °C·hr^−1^, *p* = 0.04], while nitrate ingestion showed no effect [EMM; placebo, − 0.33 (− 0.45 to − 0.20) °C·hr^−1^ vs. nitrate, − 0.36 (− 0.51 to − 0.21) °C·hr^−1^, *p* = 0.5] (Fig. 4). As a result, the delta reduction in rectal temperature at 45 min was significantly greater during hypoxia compared to normoxia trials [EMM; normoxia, − 0.25 (− 0.32 to − 0.18) °C vs. hypoxia, − 0.29 (− 0.38 to − 0.21) °C, *p* = 0.03] (Fig. 5d), while no differences were observed in the 45 min delta values between nitrate and placebo conditions. [EMM; placebo, − 0.27 (− 0.37 to − 0.18) °C vs. nitrate, − 0.28 (− 0.36 to − 0.20) °C, *p* = 0.7]. These observations for rectal temperature were reversed at the point of shivering onset; the delta reduction in rectal temperature was greater during nitrate compared to placebo trials at shivering onset [EMM; placebo, − 0.14 (− 0.24 to − 0.07) °C vs. nitrate, − 0.25 (− 0.39 to − 0.10) °C, *p* = 0.04] (Fig. 5c), while no effect was observed for hypoxia compared to normoxia [EMM; normoxia, − 0.20 (− 0.32 to − 0.07) °C vs. hypoxia, − 0.19 (− 0.29 to − 0.10) °C, *p* = 0.9). No interaction was observed between hypoxia and nitrate ingestion on either core-cooling rate or delta changes in rectal temperatures (*p* > 0.5). No main effects of hypoxia or nitrate ingestion were observed on mean skin temperature, assessed either relative to shivering onset or at the fixed 45 min time point (*p* > 0.1) (Fig. 5a, b). This observation did not differ when skin temperature sites were considered independently or in regionally (*p* > 0.3). Again, no correlation was observed between either peripheral oxygen saturation or plasma [NO_2_^−^] and thermal metrics (p > 0.06).

### Secondary variables

Secondary data are presented in Table 1. Assessed as the mean value between 10 min and shivering onset, hypoxia significantly increased heart rate and significantly decreased mean arterial pressure and peripheral oxygen saturation (main effects: heart rate, *p* = 0.02; mean arterial pressure, 0.02; peripheral oxygen saturation, *p* < 0.001). These observations were mirrored when assessed between 10 and 45 min (main effects: heart rate, *p* = 0.01; mean arterial pressure, 0.01; peripheral oxygen saturation, *p* < 0.001). No main effects of nitrate ingestion were observed across these secondary variables (*p* > 0.2). No effects of either hypoxia or nitrate ingestion were observed on expired respiratory variables, EMG or MMG across the time period relative to shivering onset or the fixed time-window (*p* > 0.2).

**Table 1:** Physiological variables in response to whole-body cooling with independent and combined hypoxia and acute inorganic nitrate ingestion

	NM_PLC	HYP_PLC	NM_NTR	HYP_NTR	Effects
*Relative to shivering onset*					
Heart rate (beats·min^−1^)	63 ± 15	67 ± 16	61 ± 16	66 ± 14	*Hyp* *Hyp* *Hyp*
Mean arterial pressure (mm Hg)	94 ± 5	92 ± 5	92 ± 9	89 ± 7	
Peripheral oxygen saturation (%)	99 ± 1	88 ± 5	99 ± 1	87 ± 4	
Respiratory rate (breaths·min^−1^)	13 ± 3	15 ± 4	14 ± 4	12 ± 3	
Minute ventilation (L·min^−1^)	9.8 ± 3.6	12.4 ± 5.0	11.3 ± 3.6	11.2 ± 3.5	
Exhaled CO_2_ (mL·min^−1^)	272 ± 95	297 ± 89	283 ± 65	291 ± 83	
O_2_ Consumption (mL·min^−1^)	345 ± 103	369 ± 136	360 ± 93	356 ± 121	
Electromyography (µV)	7.2 ± 7.4	6.3 ± 7.3	6.6 ± 5.4	6.9 ± 5.2	
Mechanomyography (m·s^2^)	0.66 ± 0.39	0.72 ± 0.46	0.63 ± 0.17	0.65 ± 0.43	
*Fixed time-window*					
Heart rate (beats·min^−1^)	65 ± 15	68 ± 17	61 ± 11	73 ± 16	*Hyp* *Hyp* *Hyp*
Mean arterial pressure (mm Hg)	94 ± 6	91 ± 5	92 ± 8	89 ± 8	
Peripheral oxygen saturation (%)	99 ± 1	88 ± 4	99 ± 1	87 ± 4	
Respiratory rate (breaths·min^−1^)	14 ± 3	15 ± 4	14 ± 3	12 ± 3	
Minute ventilation (L·min^−1^)	10.8 ± 3.9	13.6 ± 5.3	11.7 ± 3.6	11.7 ± 3.4	
Exhaled CO_2_ (mL·min-1)	302 ± 108	344 ± 122	308 ± 70	319 ± 108	
O_2_ Consumption (mL·min^−1^)	358 ± 114	389 ± 142	373 ± 94	365 ± 118	
Electromyography (µV)	8.7 ± 7.9	8.3 ± 7.8	8.2 ± 6.2	7.9 ± 5.7	
Mechanomyography (m·s^2^)	0.73 ± 0.44	0.83 ± 0.47	0.65 ± 0.17	0.71 ± 0.42	

***NM_PLC***, normoxia (*Fi*O_2_, 0.209) with placebo ingestion **(**0.003 mmol NO_3_^−^**)**; ***HYP_PLC***, hypoxia (*Fi*O_2_, 0.130) with placebo ingestion; ***NM_NTR***, normoxia with nitrate ingestion **(**13 mmol NO_3_^−^**)**; ***HYP_NTR***, hypoxia with nitrate ingestion. Variables were assessed as a mean value across both a fixed 10 min to 45 min time-window, shared by all participants and trials, and an individualised time-window between 10 min and shivering onset. Data are mean ± SD. Main effects (hypoxia and nitrate) and interactions (hypoxia x nitrate) assessed via two-way repeated-measures ANOVA (*α* = 0.05)

## Discussion

This study assessed the impact of independent and combined normobaric hypoxia and acute dietary nitrate ingestion on shivering thermogenesis and vascular control in response to acute whole-body cooling. The principle findings of this study were: (1) normobaric hypoxia (F_i_O_2,_ 0.130) expedited shivering thermogenesis onset time during whole-body cooling via mechanisms mediated primarily by increased cutaneous vasodilatation and peripheral heat loss, instead of a change in the central thermoeffector thresholds (i.e., altered afferent thermosensitivity or efferent drive). (2) Conversely, acute nitrate ingestion (13 mmol NO_3_^−^) increased the onset time of shivering thermogenesis during whole-body cooling via centrally mediated alterations in the thermoeffector thresholds, independent of vascular perfusion and peripheral heat exchange. (3) No statistical interaction was observed between hypoxia and acute nitrate ingestion on autonomic thermoeffector responses to the cold. Two hypotheses were addressed through this investigation:

***Hypothesis 1:*** *Over 45 min, hypoxia would blunt peripheral vasoconstriction, leading to an increase in skin blood flow and rate of heat loss compared to normoxia, with this response synergised following concomitant nitrate ingestion.* Consistent with previous work (Simmons et al. 2010; Arnold et al. 2020b), hypoxia blunted cutaneous vasoconstriction in response to the whole-body cold stress. As conjectured, hypoxia increased cutaneous blood flow and reduced mean arterial pressure, with a net effect of increased CVC. When combined with cold, hypoxia increased core cooling and peripheral heat loss compared to cold exposure in normoxia. This finding agrees with previous observations of Cipriano and Goldman (1975), Robinson and Haymes (1990), and Johnston et al. (1996), but not those of Blatteis and Lutherer (1976) and Simmons et al (2011), where no differences in core temperature or core-cooling rates were observed with combined cold hypoxia relative to cold alone. Differences across studies might be due in part to the duration and magnitude of hypoxic exposure and/or the cooling stimulus imposed. For example, it is possible that a prolonged exposure to hypoxia, as documented in the fieldwork by Blatteis and Lutherer (1976)—i.e., > 6 h including transport to high altitude—permitted core temperature to stabilise, and thermogenesis to resolve differences between normoxia and hypoxia.

In addition to hypoxia, hypocapnia associated with hypoxic hyperventilation also appears to modulate thermoregulation, as evidenced in cats (Gautier et al. 1989). However, while end-tidal CO_2_ partial pressure was not assessed within the current study, no main effect was observed for hypoxia on either respiratory rate, minute ventilation, or exhaled CO_2_, such as hypoxia-induced hyperventilation, and thus, hypocapnia appears to be negligible. The interaction of hypocapnia with the current findings warrants further investigation.

No independent main effect was observed for nitrate ingestion on cutaneous vascular or thermal metrics compared to a placebo. This null effect of nitrate on CVC opposes observations of vasodilatation previously reported (Keen et al. 2015; Levitt et al. 2015), while the authors of both studies ascribe the observed changes in CVC to changes in mean arterial pressure and not skin blood flow per se. Along with the observations of the present study, it is therefore unlikely that acute nitrate ingestion independently alters peripheral heat exchange, given the absence of altered skin perfusion across studies. Interestingly, no mechanistic interaction was observed between hypoxia and nitrate ingestion. This result is particularly interesting given that the reduction of NO_2_^−^ to NO is potentiated in acidosis and hypoxia (Modin et al. 2001; Castello et al. 2006; Van Faassen et al. 2009). However, the definitive contribution of NOS-dependent and NOS-independent NO synthesis to cutaneous vascular tone in hypoxia remains unclear. Indeed, while oxygen is required for NOS-derived NO (Bredt 1999), compared to other isoforms, endothelial NOS (eNOS) has the lowest Michaelis constant (highest sensitivity) for oxygen (Stuehr et al. 2004). As such, the potential for eNOS to contribute to NO synthesis in hypoxia remains likely up until extreme hypoxia or even anoxia. Furthermore, while plasma [NO_2_^−^] was elevated to a similar extent during nitrate trials, it is not known how and where this NOS-independent NO substrate was used during hypoxic-cold exposure. Interestingly, given that oral mucosa serves as a primary medium for the exogenous reduction pathway of dietary NO_3_^−^ (Granli et al. 1989), and evidence is presented for increased salivary flow rate in the cold (Walsh et al. 2002; Kariyawasam and Dawes 2005; Elishoov et al. 2008), future work should explore whether the current findings remain consistent with prolonged hypoxic-cold exposure.

***Hypothesis 2:*** *At shivering onset, no difference in skin or core temperature would be observed between hypoxia and normoxia, yet faster heat loss with hypoxia would result in a temporally earlier shivering onset, with this response further synergistically accelerated following concomitant nitrate ingestion*. As conjectured, the thermoeffector threshold for shivering thermogenesis did not differ between hypoxia and normoxia, observed as a matched rectal temperature at the point of shivering onset. Yet, as a result of greater heat loss during hypoxia, with matched oxygen uptake, the threshold was attained more rapidly in hypoxia compared to normoxia. To date, it is unclear whether humans, like several smaller mammals, experience hypoxic anapyrexia, mediated by a central resetting of the thermogenic threshold. Johnston and colleagues (1996) observed a significant reduction in the oesophageal temperature at which shivering occurs (− 0.19 °C) in hypoxia, while the observations of the current study do not support this finding. Given the similarity in the hypoxic dose and also lack of hypoxic hyperventilation between both studies, variability in the analytical method used to determine shivering onset might better explain differences between studies. To minimise error in the present study, multiple metrics were used to quantify the shivering onset inflection point across trials.

Independently, nitrate ingestion delayed the onset of shivering thermogenesis. Given that no significant differences were observed in the core cooling or peripheral heat exchange mechanisms with nitrate ingestion, this delay in shivering onset can only be attributed to a central resetting of the thermogenic thermoeffector threshold, evidenced by a lower rectal temperature at the point of onset. While research shows that peripheral administration of an NO donor can enhance skin blood flow (Johnson et al. 2014), the administration of NOS inhibitors intracerebroventricularly have also shown to reduce sympathetic brown adipose tissue thermogenesis in a number of small mammals (Gerstberger 1999). In this context, NO likely acts as a key modular of neuronal thermoregulatory activity, signifying that locally acting NO may change thermal balance in opposing directions depending on the thermoeffector involved. Note, whether nitrate ingestion can increase NO bioavailability in the brain has yet to be empirically determined. Yet, given that NO is a mediator in the opening of the blood–brain barrier (Lawther et al. 2011), it is possible that NO itself permits other molecules such as nitrate and nitrite to transcend the barrier. Furthermore, as sialin, the nitrate transporter, has been evidenced in brain tissue (Qin et al. 2012), if nitrate can cross the barrier, it could have a physiological effect.

### Considerations

Despite a significant change in both CVC and rectal temperature with hypoxia, no difference was observed in mean skin temperature, as would be expected with greater peripheral heat loss. Though attention was paid to ensuring a robust estimate of skin temperature, using a 14-site model with calibrated thermistors, a possible explanation for the non-significant skin temperature finding might be measurement error. Indeed, when the temperature gradient within the measurement system is high; for example, skin temperature (25–35 °C) vs. low environmental temperatures (10 °C), the potential for measurement error, and variability increases (MacRae et al. 2018). An increase in variability directly reduces the potential for observing statistically significant differences between conditions. A second consideration is whether an extension of the current work sampled across a heterogenous cohort may unveil interesting correlations between physiological metrics and changes in plasma [NO_2_^−^]. This may have been otherwise masked by the homogenous nature of the participants recruited herein—i.e., healthy, young, active university students. Finally, given that maximal CVC was not established for the purpose of normalisation, data should be interpreted with care. The authors acknowledge that, even when care is taken to measure cutaneous blood flow at the same location for repeated measurements, the precise location and thus exact local vasculature are likely not the same. This spatial variability was partly mitigated through the use of an integrated multi-array laser Doppler probe in the current investigation (Cracowski and Roustit 2020). Furthermore, the authors believe that the strengths of the statistical design (repeated-measures ANOVA) increase the credibility of each conclusion, given that variances across conditions are rigorously accounted for. For example, a significant main effect of hypoxia on CVC was considered against the variances in CVC across all four visits.

## Conclusion

To date, it is unclear whether humans exhibit suppressed autonomic thermoeffector responses to the cold with hypoxia, and whether this is modulated by nitric oxide availability, either peripherally or centrally. The current investigation presents key original evidence for a hypoxic reduction in the onset time of shivering thermogenesis during whole-body cooling, via mechanisms mediated primarily by increased cutaneous vasodilatation and peripheral heat loss, instead of a change in the central thermoeffector thresholds. This response was not synergised with increased nitric oxide availability via dietary nitrate supplementation. On the other hand, acute nitrate ingestion independently increased the onset time of shivering thermogenesis during whole-body cooling via mechanisms mediated primarily by a change in the central thermoeffector thresholds, independent of vascular perfusion and peripheral heat exchange. These novel findings increase our basic understanding of how systemic hypoxia modulates thermoregulatory responses, and its independence from any increases in nitric oxide bioavailability afforded by dietary nitrate supplementation.

## Abbreviations

CI: Confidence interval
CNS: Central nervous system
CVC: Cutaneous vascular conductance
EMG: Electromyography
EMM: Estimated Marginal Means (EMM)
MMG: Mechanomyography
NO: Nitric oxide
NO_2_^−^: Nitrite
NO_3_^−^: Nitrate
NOS: Nitric oxide synthase
S_p_O_2_: Peripheral oxygen saturation
USG: Urine specific gravity
V̇O_2_: Oxygen uptake
